# Regional brain metabolism in schizophrenia: An FDG-PET study

**DOI:** 10.4103/0019-5545.31577

**Published:** 2006

**Authors:** R. Seethalakshmi, S.R. Parkar, N. Nair, S.A. Adarkar, A.G. Pandit, S.A. Batra, N.S. Baghel, S.H. Moghe

**Affiliations:** * Research Associate, Department of Psychiatry, K.E.M. Hospital, Mumbai; ** Professor and Head, Department of Psychiatry, K.E.M. Hospital, Mumbai; **** Lecturer, Department of Psychiatry, K.E.M. Hospital, Mumbai; ****** Research Associate, Department of Psychiatry, K.E.M. Hospital, Mumbai; *** Head, Radiation Medicine Centre, Bhabha Atomic Research Centre, Mumbai; ***** Resident Medical Officer, Radiation Medicine Centre, Bhabha Atomic Research Centre, Mumbai; ******* Scientific Officer, Radiation Medicine Centre, Bhabha Atomic Research Centre, Mumbai; ******** Scientific Officer, Radiation Medicine Centre, Bhabha Atomic Research Centre, Mumbai

**Keywords:** Brain metabolism, schizophrenia, FDG-PET

## Abstract

**Background:**

Recent technological advances have established beyond any doubt the biological nature of schizophrenia. Functional neuroimaging using FDG-PET forms an important technique in understanding the biological underpinnings of psychopathology of schizophrenia.

**Methods:**

Eighteen male patients diagnosed as having schizophrenia and having active psychosis as determined by PANSS were subjected to FDG-PET scanning under resting conditions. The glucose uptake in selected regions of interest was studied across the spectrum of schizophrenia.

**Results:**

Chronicity and severity of illness did not influence cerebral glucose metabolism. Participants with negative schizophrenia had significantly decreased metabolism in all regions of the brain as compared to the positive type. The positive syndrome of schizophrenia was associated with significantly increased glucose metabolism in the medial temporal regions, basal ganglia and left thalamic regions. Hypometabolism was also noted in the cerebellum.

**Conclusion:**

While a number of brain areas can be identified as potential causative regions and hypotheses regarding putative mechanisms can be formed, the considerable heterogeneity of schizophrenia poses a great challenge in the precise delineation of the disease process.

## INTRODUCTION

In 1990, Ron and Harvey^1^ commented: ‘20 years ago, the principal challenge for schizophrenia research was to gather objective scientific evidence that would implicate the brain. That challenge no longer exists.’ Indeed, the biological aetiology of schizophrenia has been established beyond any doubt. However, the considerable heterogeneity of this disorder has made the precise delineation of specific biological areas a Herculean task.

One modality that is rapidly gaining popularity in functional neuroimaging is Positron Emission Tomography (PET). PET techniques employ positron-emitting radiotracers to measure localized changes in cerebral blood flow and glucose meta-bolism. One of the earliest studies of PET in schizophrenia dates back to the year 1974 when Ingvar *et al.*^2^ compared whole brain metabolic rates in individuals with schizophrenia and normal controls. Since then a number of studies^3^–^9^ employing refined methodological parameters have been successful in establishing differences in cerebral metabolic patterns between individuals with schizophrenia and normal controls, between the different types of schizophrenia and among the different symptom profiles. Recent PET studies have also suggested cerebellar involvement as a part of the cognitive dysmetria of schizophrenia.^10^ It has been suggested that abnormal circuitry or dysconnection syndromes could be the primary pathology in schizophrenia.^10^ We aimed to examine the regional cerebral glucose metabolism in individuals with schizophrenia and to explore the association of the same with the illness profile of the patients.

## METHODS

The study was conducted by the psychiatry department of a tertiary care hospital in collaboration with the Radiation Medicine Centre, Bhabha Atomic Research Centre, Mumbai. Approval for the study was obtained from the Institutional Review Boards of both collaborating institutes. Eighteen males with a confirmed diagnosis of schizophrenia (ICD-10)^11^ and scoring between 70 and 120 on the Positive and Negative Symptom Scale (PANSS)^12^ were recruited from the outpatient psychiatry unit. Patients with other comorbid Axis I psychiatric diagnoses, comorbid substance abuse or dependence except nicotine dependence, mental retardation on clinical evaluation, past or current history of any neurological illness, or any other medical illness and with blood sugar >120 mg/dl were excluded from the study. Participants were informed about the purpose and nature of the study and a written informed consent was taken from both the participant and the caregiver in the presence of a impartial witness. Participants were subsequently administered a semi-structured questionnaire on sociodemo-graphic profile, family history, handedness and duration and course of schizophrenia. Participants also underwent a detailed neurological and physical examination.

FDG-PET scan of the brain was carried out within 24 hours of the PANSS assessment. Participants were advised overnight fasting and their blood sugar levels were checked prior to the scan. An average dose of 200 MBq (160–230) of F-18, 2-fluoro, 2-deoxy-glucose (F18-FDG) was injected. Participants were subsequently asked to rest in a quiet, well-lit room and were asked to refrain from talking. Their eyes remained open and ears were unoccluded. Acquisition was carried out 30 minutes after the injection. Positioning was achieved with the help of LASER align lights. The head was secured with restraints to minimize artifacts due to movement. The distribution of cerebral metabolism of glucose was examined using a GE advance PET system scanner NXI. The scanner has a trans-axial resolution of 4.8–6.2 mm FWHM (Full Width Half Maximum) depending upon the distance from the centre and an axial resolution of 4.0–6.6 mm FWHM. Emission scans of 70 slices were obtained parallel to the cantho-meatal line from the vertex to the neck. Transmission scans were obtained for the same regions using germanium-68 (Ge-68) rod sources to carry out measured attenuation correction. The images were reconstructed using the Ordered Subsets Expectation Maximization (OSEM) algorithm. These images were reformatted and converted into 35 trans-axial slices of 4.25 mm thickness and 17 trans-axial slices of 8.5 mm thickness. The former were used for qualitative analysis and the latter for quantitative analysis. Regional glucose metabolism was examined in 14 predetermined Regions of Interest (ROIs): elliptical ROIs for cortical and subcortical structures and circular ROIs for cerebellar hemispheres. The size of ROI for cortical and subcortical areas was kept at 6.31 sq. cm; the pixels varied from 46 to 52. The ROI for cerebellum was 14.03 sq. cm and the pixels varied from 96 to 104.

### Analysis

The data were analysed using the Statistical Package for Social Sciences 11.0 (SPSS, Chicago, IL, USA). Positive and Negative type was determined based on the composite score of PANSS (Positive subscale–Negative subscale).^12^ Positive and negative components of schizophrenia were calculated as described by Buchsbaum *et al.*^13^ (Positive component is the sum of scores on the items of unusual thought content, delusions, grandiosity, lack of judgement and insight and hallucinatory behaviour. Negative component is the sum of scores on the items of emotional withdrawal, passive/apathetic social withdrawal, lack of spontaneity and flow of conver-sation, blunted affect, poor rapport, poor attention, active social avoidance, motor retardation, disturbance of volition, mannerisms, and posturing.)

For the purpose of analysis, the slice of the brain through the basal ganglia was taken as the reference slice. One slice above the basal ganglia and one below were checked for maximum activity uptake values (mUVs) for various ROIs. For the cerebellum, mid-cerebellar slice was selected. The ROIs were selected by a neurodiagnostician who was blinded to the symptom profile of the participants. ROIs considered included the following:

1. Prefrontal regions (right and left)
2. Temporal (right, left, medial and lateral)
3. Parietal (right and left)
4. Occipital (right and left)
5. Basal ganglia (right and left)
6. Thalamus (right and left)
7. Cerebellum (right and left)

Maximum regional activity uptake values (mUVs) were measured in kBq/cc. These mUVs were considered for inter-group variances in regional metabolism.

Relative regional activity (rUV) was further calculated as a ratio of maximum activity within an ROI to the average of the maximum activities in the occipital ROIs. It has been suggested that the absolute values may show individual differences and that the pattern of regional glucose activity is better represented by relative values.^13^ Occipital ROIs were selected as the normalizing factors since these ROIs displayed the highest activity of all ROIs considered in majority of the patients. The cerebellum has been used as a normalizing factor in an earlier study.^14^ However, in this study it was not considered feasible as the blinded diagnostician reported significant cerebellar metabolic changes. Besides, recent studies^9^,^10^,^15^–^17^ have also reported cerebellar changes. Relative regional activity uptake values (rUVs) were considered primarily for comparing the different ROIs.

## RESULTS

The mean age of the sample was 28.7 years (range 19–44 years; SD 6.61 years); 72% of the participants had a minimum of secondary education; 14 were unemployed; 11 participants were married; 1 participant was left-handed. History of nicotine dependence was present in 66% of the participants. Family history of schizophrenia was present in one-third of the participants (Table 1). The mean age at onset of illness was 22.7 years; average duration of illness was 5.9 years. Total PANSS scores ranged from 78 to 119 (mean 94.1). None of the participants had any abnormal movements on examination. On the composite scale, 11 participants had positive and 7 had negative schizophrenia.

**Table 1:** Sociodemographic profile of the sample

Profile		Frequency	%
Education	Graduate	2	11.1
	Illiterate	1	5.6
	Primary	2	11.1
	Secondary	13	72.2
Occupation	Service	4	22.2
	Unemployed	14	77.8
Marital status	Married	7	38.9
	Unmarried	11	61.1
Handedness	Left	1	5.6
	Right	17	94.4
Nicotine dependence	No	6	33.3
	Yes	12	66.7
Family history	No	12	66.7
	Yes	6	33.3

**Fig 1:**
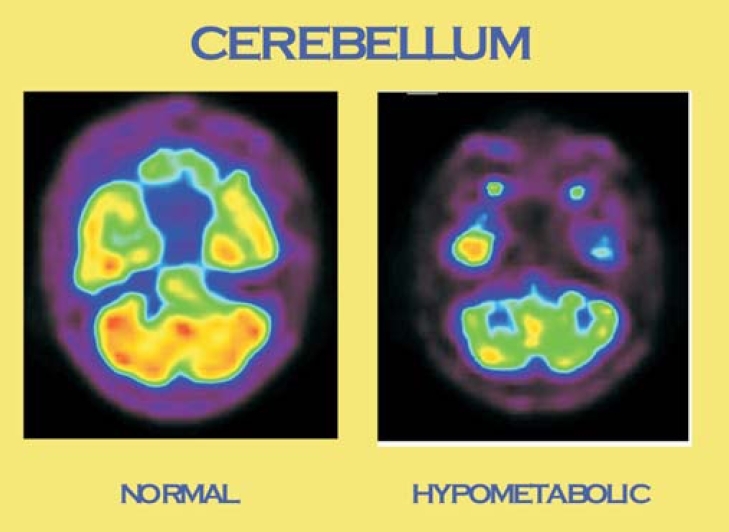
Normal cerebellar uptake and cerebellar hypometabolism observed in individuals with schizophrenia

On qualitative assessment, 4 showed a global reduction in metabolism, 5 had decreased uptake in all regions except the occipital lobes, 6 had specific regional decrease and 3 scans were nearly normal. The basal ganglia of 8 participants had increased uptake, 2 demonstrated decreased uptake independent of overall metabolism and the remaining 8 showed no significant abnormality. Cerebellar uptake was reduced in 10 of the 18 participants (Fig 1.); in 2 participants, this finding existed even in the absence of a global reduction in metabolism.

Neither the age of onset of illness nor the duration of illness showed any statistically significant relationship (p>0.05) with glucose uptake. The mUVs in all ROIs correlated positively with total PANSS scores except in the right occipital and left cerebellum, which correlated negatively. The rUVs in all ROIs except right occipital, left cerebellum and the left thalamus correlated positively with total PANSS scores; rUV in the right thalamus showed a significant positive correlation with the severity of illness on the PANSS score (p<0.05).

### Type of schizophrenia and regional glucose metabolism (Table 2)

**Table 2:** Difference between maximum cerebral glucose uptake (kBq/cc) in the two types of schizophrenia

ROI	Positive type (*n*=11)	Negative type (*n*=7)	p value
R frontal	21.96	10.72	0.010**
L frontal	22.14	11.42	0.013*
R parietal	22.37	10.70	0.006**
L parietal	22.21	11.76	0.014*
RM temporal	15.61	7.90	0.008**
LM temporal	15.27	8.25	0.012**
RL temporal	19.34	11.60	0.067
LL temporal	19.57	10.86	0.037*
R occipital	26.41	14.19	0.011*
L occipital	26.58	14.12	0.008**
R cerebellum	20.32	9.56	0.007**
L cerebellum	20.17	10.38	0.010**
R basal ganglia	23.23	12.60	0.013*
L basal ganglia	22.69	11.76	0.003**
R thalamus	19.77	11.20	0.016*
L thalamus	20.75	10.37	0.005**

** p<0.01

* p<0.05 (2-tailed significance)

Between the two types of schizophrenia (as per the composite scale), mUVs in all the ROIs were significantly lower (p<0.05) in individuals with predominantly negative symptoms as compared to participants with predominantly positive symptoms except in the right lateral temporal region. The difference in glucose uptakes was highly significant (p<0.01) in the right cortical regions (frontal and parietal), medial temporal regions bilaterally, both cerebella, left basal ganglia and left thalamus. However, when corrected for multiple comparisons, these differences did not attain significance.

### Symptom profile and regional glucose metabolism (Tables 3 and 4)

**Table 3:** Correlation between regional glucose metabolism (maximum and relative) and positive syndrome scale scores

ROI	Maximum glucose uptake (kBq/cc)		Relative glucose uptake	
		
	Pearson's coefficient	p value	Pearson's coefficient	p value
R frontal	0.466	0.051	0.380	0.119
L frontal	0.448	0.062	0.224	0.371
R parietal	0.461	0.054	0.374	0.126
L parietal	0.422	0.081	0.211	0.401
RM temporal	0.541	0.020*	0.431	0.074
LM temporal	0.481	0.043*	0.117	0.643
RL temporal	0.332	0.178	−0.165	0.513
LL temporal	0.412	0.089	0.048	0.851
R occipital	0.432	0.074	−0.183	0.467
L occipital	0.438	0.069	0.204	0.417
R cerebellum	0.423	0.080	-	-
L cerebellum	0.422	0.081	-	-
R basal ganglia	0.500	0.034*	0.104	0.683
L basal ganglia	0.529	0.024*	0.167	0.507
R thalamus	0.420	0.083	−0.024	0.926
L thalamus	0.498	0.035*	0.348	0.158

* Correlation is significant at the 0.05 level

**Correlation is significant at the 0.01 level

**Table 4:** Correlation between regional glucose metabolism (maximum and relative) and negative syndrome scale scores

ROI	Maximum glucose uptake (kBq/cc)		Relative glucose uptake	
		
	Pearson's coefficient	p value	Pearson's coefficient	p value
R frontal	−0.277	0.266	−0.143	0.570
L frontal	−0.241	0.335	0.106	0.676
R parietal	−0.300	0.226	−0.150	0.553
L parietal	−0.232	0.355	0.178	0.480
RM temporal	−0.317	0.201	−0.021	0.934
LM temporal	−0.280	0.260	0.200	0.426
RL temporal	−0.170	0.499	0.269	0.281
LL temporal	−0.211	0.400	0.055	0.827
R occipital	−0.288	0.247	0.139	0.583
L occipital	−0.279	0.263	−0.102	0.688
R cerebellum	−0.293	0.238	-	-
L cerebellum	−0.283	0.255	-	-
R basal ganglia	−0.304	0.219	0.109	0.668
L basal ganglia	−0.308	0.214	0.110	0.664
R thalamus	−0.215	0.392	0.419	0.083
L thalamus	−0.336	0.173	−0.389	0.111

*Correlation is significant at the 0.05 level

**Correlation is significant at the 0.01 level

When glucose activity uptake values in the ROIs were correlated with the symptom profile (the positive and negative syndrome scale scores), mUVs in both medial temporal regions, bilateral basal ganglia and left thalamus positively correlated with the positive syndrome score significantly (p<0.05). There was however no significant correlation (p>0.05) of the rUVs of the different ROIs with the positive syndrome score. The scores on the negative syndrome scale correlated negatively with mUVs in all ROIs, though not significantly (p>0.05); the rUVs in the right cortical regions, bilateral cerebella, and left thalamus showed a negative correlation that also was not significant (p>0.05).

## DISCUSSION

Buchsbaum and colleagues^13^ were the pioneers to suggest that the pattern of cerebral glucose utilization may be altered in patients with schizophrenia as compared to normal volunteers. Subsequently, a number of studies have reported lower whole brain metabolic rates in individuals with schizo-phrenia as compared with normal controls.^4^,^5^,^18^ A recent study has reported increased metabolism in unmedicated patients with schizophrenia.^9^ On qualitative evaluation of the scans, a global reduction in metabolism was observed in 50% (including normal occipital uptake) of the participants. Buchsbaum *et* al.^13^ also suggested that decreased cerebral glucose uptake appears early in the disease process and is not related to chronicity, symptom severity or medication exposure. Contrary to this, both Gur *et* al.^5^,^6^ and Szechtman *et al*.^19^ reported that the severity of illness and the duration of illness influence cerebral metabolism. In this study, neither severity nor chronicity of illness had any influence on cerebral glucose uptake.

However, differences in metabolism were noted between the two types of schizophrenia. The negative type of schizo-phrenia was associated with decreased metabolism in all ROIs as compared to the positive type. Decreased glucose uptake was observed in all neocortical areas of the brain (frontal, parietal, temporal and occipital regions) along with the thalamus and the limbic system in patients with the deficit type of schizophrenia as compared to patients with the non- deficit type.^20^

Positive symptoms of schizophrenia have been associated with temperolimbic dysfunctions.^21^ Maximum glucose uptake in the medial temporal regions, basal ganglia and the left thalamus showed a significant increase with increasing scores on the positive syndrome. However, rUVs that are better reflective of inter-regional differences did not demonstrate any such variations. One possible reason for the increased uptake in the basal ganglia is the possibility of greater and more vigorous neuroleptization in positive symptoms. This regional uptake also delineates the mesio-striato-thalamic circuit for goal-directed behaviour^22^ as an important pathology in positive symptoms and exemplifies schizophrenia as a ‘lateralized brain disorder’.^23^ Consistent with literature,^16^ negative symptoms, on the other hand, were associated with decreased metabolism in all ROIs.

Interest in the role of the cerebellum as a key perpetrator in the psychopathology of schizophrenia is increasingly gaining momentum. The implication that the cerebellum may be involved in the cognitive dysmetria of schizophrenia through the prefrontal–thalamic–cerebellar circuitry was proposed by Andreasen *et al*.^10^ The majority of our participants had reduced cerebellar metabolism qualitatively. Intriguingly, cerebellar hypometabolism was noted in the cerebellum in the absence of any clinically apparent symptoms or signs of cerebellar involvement.

Our study has a few limitations. The ROI method of analysis that we used relatively lacks anatomical detailing. It is possible to determine the location of an ROI but its exact relationship to individual gyri and sulci cannot be known. Due to technol-ogical constraints, we had to limit ourselves to this method of analysis. One way of overcoming this limitation is to obtain anatomically detailed images such as MRI. However, this requires co-registration that we were unable to perform. The small sample size of the study population, multiple confounding factors such as varied states of neuroleptization and multiple comparisons also warrant a careful interpretation of the results. The study also did not compare the metabolic changes with uptake values in normal controls; however, a qualitative assessment by an expert was included to compensate for the same.

Schizophrenia is considered the most intractable of mental illnesses and among the least comprehensible in terms of neurobiological mechanisms. The foremost reason for this is the heterogeneity in its phenotypic expression. Though functional imaging studies have identified abnormalities in a number of brain structures, no primary site has yet been defined. This study reflects involvement of a number of brain areas, which vary with the symptom profile. The region that emerged as a potential region for further research was the cerebellum, which indeed has been identified as an important mediator of cognition. Finally, one has to agree with Chua and McKenna^24^ that ‘schizophrenia shows complex alterations in regional patterns of activity and not a simple deficit in prefrontal function’.
